# Fatal immune-related hepatitis with intrahepatic cholestasis and pneumonia associated with camrelizumab: A case report and literature review

**DOI:** 10.1515/med-2021-0267

**Published:** 2021-04-07

**Authors:** Youwen Tan, Yun Ye, Li Chen

**Affiliations:** Department of Hepatology, The Third Hospital of Zhenjiang Affiliated Jiangsu University, No. 300, Daijiamen, Runzhou Distinct, Zhenjiang 212003, China

**Keywords:** camrelizumab, immune-related hepatitis, intrahepatic cholestasis, immune-related pneumonia

## Abstract

Camrelizumab (SHR-1210), a human monoclonal antibody against programmed death receptor 1 (PD-1), blocks the binding of PD-1 to PD-L1, consequently inhibiting immune system evasion by tumor cells. A 65-year-old man underwent radical esophagectomy 5 months ago following the diagnosis of esophageal cancer by gastroscopy. Approximately 40 days later, capecitabine was administered at a dosage of 1.5 g Po bid for 14 days, and anti-PD-1 (camrelizumab 200 mg) was administered twice. Around 20 days later, abnormal liver function was detected. He received a diagnosis of drug-induced liver injury. Chest computed tomography scanning revealed interstitial inflammatory lesions in both lower lungs. Liver biopsy revealed immune injury with ductopenia. Therefore, the diagnosis was revised as immune-related pneumonia and hepatitis associated with camrelizumab. The treatment regimen of methylprednisolone was adjusted to 40 mg/day and gradually increased to 80 mg/day. Mycophenolate mofetil was administered at a dose of 2 g/day. Consequently, chest tightness and shortness of breath resolved, and pulmonary inflammation improved. However, jaundice did not improve and continued to exacerbate. The last measured prothrombin time was 41 s, prothrombin activity was 19%, and the international normalized ratio was 4.03. The cause of death was diagnosed as liver failure, cardiopulmonary failure, and septic shock.

## 1 Introduction

Programmed death receptor 1 (PD-1), also known as CD279, is an important immunosuppressive molecule. It regulates the immune system and promotes self-tolerance by downregulating the response of the immune system to human cells and inhibiting T cell inflammatory activity. Immunomodulation via targeting PD-1 is of great significance in tumors, infections, and autoimmune diseases as well as organ transplantation survival. PD-1/PD-L1 antibody has been widely used in a variety of tumors and demonstrates very good clinical effects. However, in recent years, immune-related adverse events (irAEs) associated with anti-PD-1 or anti-PD-L1 agents have been reported, indicating that the immune system is over-activated. With the widespread application of immune checkpoint inhibitors (ICIs), clinicians encounter an increasing number of patients with irAEs. Hepatitis, pneumonia, colon cancer, heart inflammation, and nervous system inflammation are the common irAEs. There is no effective treatment for immune myocarditis, and the mortality rate is as high as 46% [1]. De Martin et al. reported that liver injury due to immunotherapy is uncommon, and severe liver injury is rare in patients undergoing immunotherapy [2].

Camrelizumab (SHR-1210), a human monoclonal antibody against PD-1, blocks the binding of PD-1 to PD-L1, thus inhibiting immune system evasion by tumor cells. It shows a high affinity for PD-1 (KD = 3.31 nmol/L) and a high occupancy rate on circulating T lymphocytes (85% at a dose of 200 mg) [3]. Phase I clinical studies of camrelizumab have shown very good safety [4,5]. Camrelizumab showed good activity and completeness in advanced hepatocellular carcinoma and gastrointestinal cancer [6,7]. In addition, it is an effective drug for second-line tumor treatment [8]. Here, we report for the first time a case of fatal immune-related hepatitis with intrahepatic cholestasis, ductopenia, and pneumonia associated with camrelizumab that finally progressed into liver failure complicated with cardiopulmonary failure, resulting in death.

## 2 Case report

**Figure 1:**
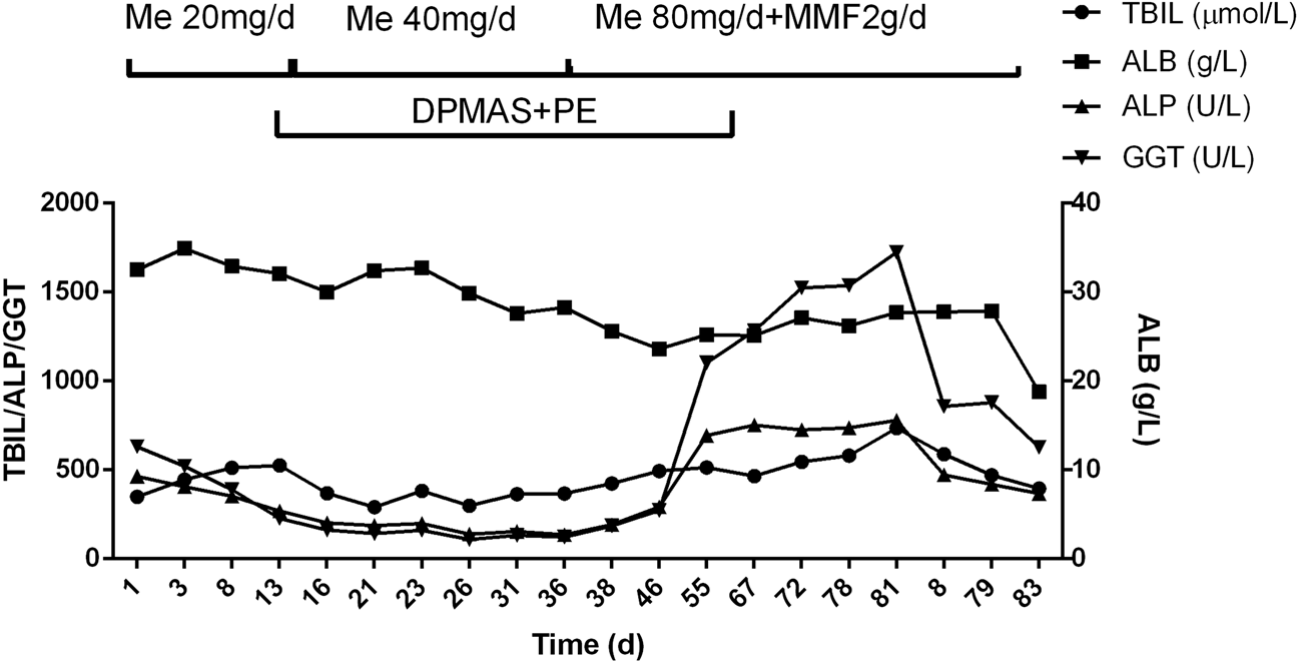
Liver function changes and treatment flow chart.

**Figure 2:**
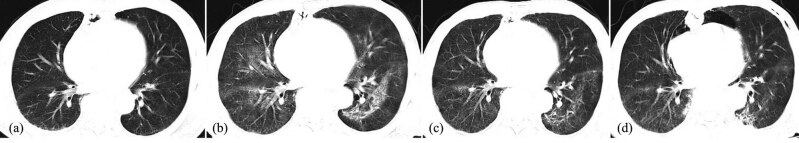
Changes in lung computed tomography scan. (a) At the beginning of the disease, the two lower lungs showed mild inflammation and exudation. (b) One month later, lung exudation aggravated; the clinical symptoms were chest tightness and shortness of breath. (c) Two weeks after treatment with methylprednisolone (1 mg/kg/day), the lesions were markedly absorbed, and the symptoms disappeared. (d) In the last week, pulmonary inflammation exacerbated, and mediastinal pneumothorax (arrow) appeared.

**Figure 3:**
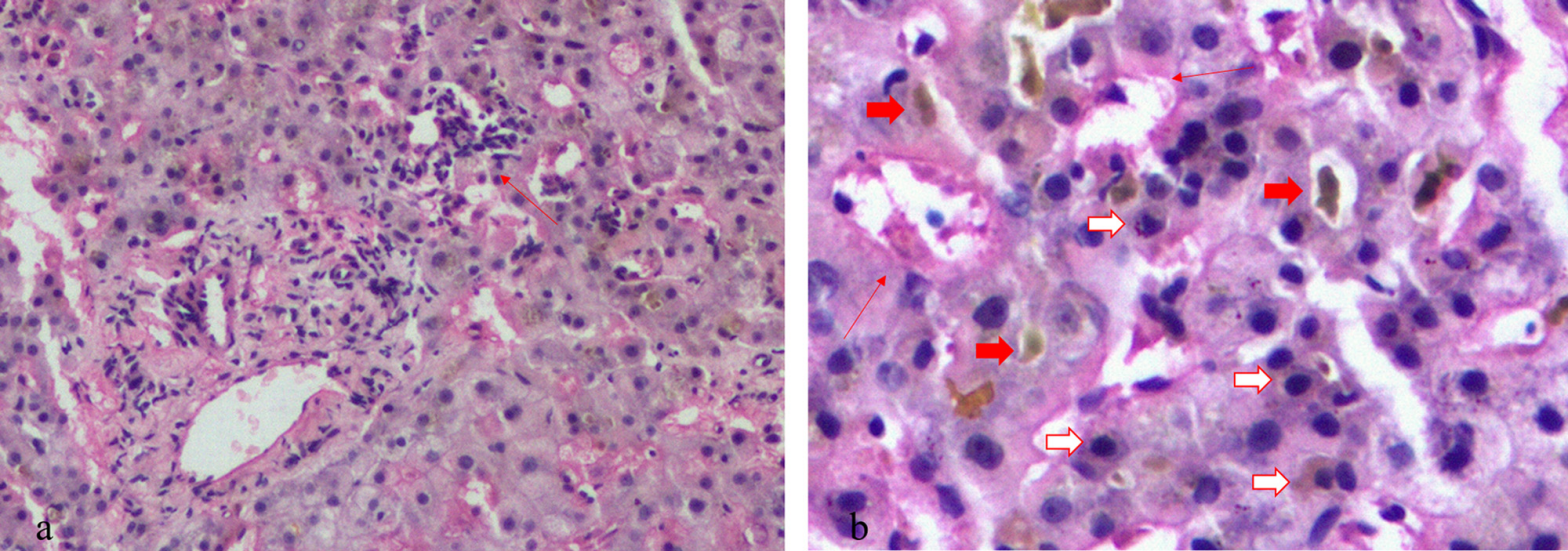
Pathological changes in a liver puncture. (a) Histological features of the liver were mainly lobular inflammation (arrow), with ductopenia, cholestasis of the capillary bile duct, and no obvious liver fibrosis (100, hematoxylin-eosin staining). (b) Panlobular hepatitis with lymphoplasmacytic infiltration (white arrow), hemorrhage of sinus endothelium (arrow), and cholestasis of capillary bile duct (red arrow) (200, hematoxylin-eosin staining).

**Figure 4:**
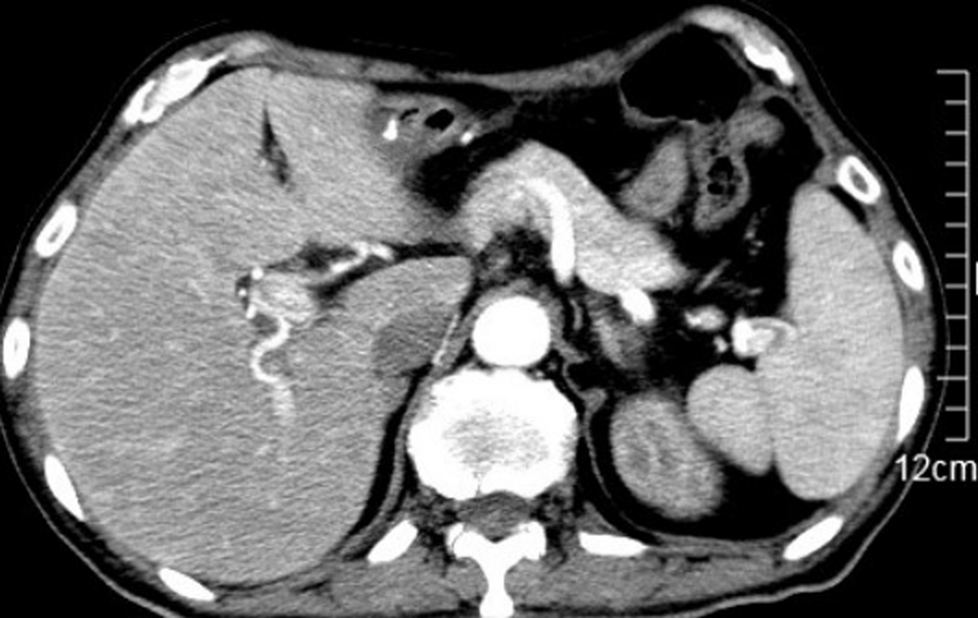
Contrast-enhanced abdominal computed tomography showing heterogeneous enhancement of liver density.

A 65-year-old man underwent radical esophagectomy 5 months ago following the diagnosis of esophageal cancer by gastroscopy. The postoperative pathology was well-differentiated squamous cell carcinoma of the esophagus, involving the adventitia and local foci around the esophagus. Approximately 40 days later, mediastinal lymph node metastasis was observed. Capecitabine was administered at a dosage of 1.5 g per os, twice daily for 14 days. At that time, the patient’s liver and kidney function tests were normal, and 1 week later, an anti-programmed death-ligand 1 agent (camrelizumab 200 mg) was administered twice with a 2-week interval between each dose. Around 20 days later, abnormal liver function was detected: total bilirubin (TBIL), 52.2 µmol/L; direct bilirubin (DBIL), 38.8 µmol/L; alanine aminotransferase (ALT), 176.7 U/L; aspartate aminotransferase (AST), 250.5 U/L; alkaline phosphatase (ALP), 336 U/L; and γ-glutamyl transpeptidase (GGT), 638 U/L. He received a diagnosis of drug-induced liver injury (DILI). Approximately 1 week later, his ALT and AST levels decreased rapidly, and TBIL continued to deteriorate (Figure 1). Considering the possibility of intrahepatic cholestasis, the physician added ursodeoxycholic acid (25 mg/kg/day) and methylprednisolone (20 mg/day). However, the cholestasis did not improve notably. Then, plasma exchange (PE) and a double plasma molecular absorption system (DPMAS) for blood purification were added, and cholestasis improved within a short time (Figure 1). During this period, except for yellow skin and dark urine, the patient had no other symptoms. Nonetheless, chest tightness and shortness of breath developed after 1 month of hospitalization. Chest computed tomography (CT) scanning revealed interstitial inflammatory lesions in both lower lungs (Figure 2). Liver biopsy revealed nonspecific immune injury (Figure 3). The doctor revised the diagnosis as immune-related pneumonia and hepatitis associated with camrelizumab. The treatment regimen of methylprednisolone was adjusted to 40 mg/day and gradually increased to 80 mg/day and mycophenolate mofetil 2 g/day was also added. Consequently, chest tightness and shortness of breath resolved, and pulmonary inflammation improved (Figure 2). Nevertheless, jaundice did not improve and continued to exacerbate. During the last 2 weeks of hospitalization, the patient showed symptoms such as high fever, decreased appetite, vomiting, and diarrhea. He was treated with human serum albumin (0.5–1.0 g/day), human hemoglobin (1.0–1.5 g/day), cefoperazone sulbactam (2 g, q12h), and caspofungin (50 mg). The last measured prothrombin time was 41 s, prothrombin activity was 19%, and the international normalized ratio was 4.03. The cause of death was diagnosed as liver failure, cardiopulmonary failure, and septic shock. The serum markers of hepatitis A–E were negative; nuclear antibody, mitochondrial antibody, liver, and kidney microsomal antibody, and anti-liver soluble antigen-antibody were negative; and immunoglobulin G value was 8.93 g/L (<16 g/L). Obstructive jaundice was excluded by multiple imaging examinations. Contrast-enhanced abdominal CT showed heterogeneous enhancement of liver density (Figure 4).

## 3 Discussion

The toxic effects caused by ICIs can affect all human organs. The underlying mechanism involves the activation of self-reactive T cells, which damage human tissues. The common organs affected by ICIs include the colon, liver, lung, and pituitary. Colon injury mainly results in diarrhea and abdominal pain, liver injury causes abnormal liver function, and lung injury leads to interstitial pneumonia. Although the pituitary, heart, and other organs are often not damaged, damage to these organs results in a very high mortality rate. The mortality rate of immune myocarditis is as high as 46% [1].

In a meta-analysis performed by searching the VigiBase database (http://www.vigiaccess.org/) [9], a total of 613 cases of fatal toxicity events related to ICIs were collected from 2009 to January 2018, of which 124 cases had hepatitis manifestations: 31 cases (31/193) caused by ipilimumab; 74 cases (74/333) caused by anti-PD-1/PD-L1; and 19 cases (19/22) caused by the mixture of two drugs.

In a study by De Martin et al., of the 536 patients treated with anti-PD-1/PD-L1 or CTLA-4 immunotherapeutics [2], 16 patients developed grade ≥3 hepatitis. Nine patients were treated with anti-PD-1/PD-L1. Liver tissue injury associated with CTLA-4 mAb was characterized by granulomatous hepatitis, fibrosis, and central venous dermatitis. The liver histological damage associated with anti-PD-1/PD-L1 treatment was characterized by inflammatory injury dominated by lobulitis. Six patients recovered spontaneously. Seven patients received methylprednisolone 0.5–1 mg/kg/day, two patients received 0.2 mg/kg/day, and one patient received 2.5 mg/kg/day of immunosuppressive drugs. Liver failure did not occur in any patient. Three of them were treated again because of the recurrence of hepatitis. De Martin et al. reported that immune-related hepatitis damage caused by ICIs is rare (3.5%). Liver biopsy helped identify whether the damage was caused by anti-PD-1/PD-L1 or CTLA-4 mAb. The liver damage improved spontaneously in some patients, while in some patients corticosteroid treatment was required. These findings indicate that a biopsy could help confirm liver damage and severity.

The currently available pathological descriptions of liver injury induced by ICIs are based on the findings from case reports [10,11,12,13]. Nonspecific immune injury is the main feature of liver injury due to ICIs, and the main lesion area is lobules rather than the portal area. Clinicopathological findings of ipilimumab-associated hepatitis were severe panlobular hepatitis and perivenular infiltrate with endothelialitis in two cases – in one case, it was mainly bile ductular proliferation, with mixed portal infiltrate around the bile ducts [14]. The pathological features in our patient were lobulitis with lymphoplasmacytic infiltration, hemorrhage of sinus endothelium, and obvious inflammation.

In a parallel-group, randomized, phase 2 trial of camrelizumab performed in patients with advanced hepatocellular carcinoma, 12 patients developed abnormal liver function, of which six (3%) had grade 1–2; five (2%) had grade 3, and one had grade 4 and died [8].

Regarding the management of immune-related hepatitis injury due to ICIs, most patients with grade 1–2 AEs can recover by themselves after withdrawal of the drug. Immunosuppressants are required for the management of liver injury above grade 3. Methylprednisolone at a dose of 0.5–2.0 kg/day is recommended for grade ≥3 liver injury, and cytoinhibitors are added according to the patient’s condition [13,15].

Our patient was initially diagnosed with DILI. Interstitial inflammation of the lungs and immune inflammation of the liver were the main manifestations. The diagnosis was modified as immune injury related to ICIs. The obvious abnormality in our patient’s liver function was hyperbilirubinemia, accompanied by an increase in the choledochal enzymes, alkaline phosphatase, and gamma-glutamyl transferase, which are manifestations of intrahepatic cholestasis. Treatment with ursodeoxycholic acid did not result in any obvious improvements. The combination of double plasma molecular adsorption system and plasma exchange treatment can improve hyperbilirubinemia through physical adsorption and plasma exchange [16]. This method is widely used in China [17,18]. Patients who undergo this treatment show a marked improvement in their hyperbilirubinemia. However, liver pathology has shown that non-specific immune injury to the liver is accompanied by ductopenia (which is a possible characteristic of immune checkpoint inhibitors) that determines whether the jaundice is difficult to manage. Corticosteroids and immunosuppressants are measures that are used to control severe liver injury; however, their effects are often unsatisfactory [19]. Although increasing the use of immunosuppressants improved pulmonary inflammatory lesions for a certain time, opportunistic infection gradually appeared. Sepsis developed into liver failure, cardiopulmonary failure, septic shock, and multiple organ failure. Based on these findings, it can be suggested that there is no specific treatment for immune injury related to ICIs.

In conclusion, we reported on a case of immune-associated liver injury and immunological pneumonia that was mainly characterized by intrahepatic cholestasis and pathologically indicated intrahepatic bile duct deficiency. A variety of treatments did not stop the disease progression and the patient still developed multiple organ failures and, ultimately, died.
